# Simplified Admix Archaeal Glycolipid Adjuvanted Vaccine and Checkpoint Inhibitor Therapy Combination Enhances Protection from Murine Melanoma

**DOI:** 10.3390/biomedicines7040091

**Published:** 2019-11-23

**Authors:** Felicity C. Stark, Gerard Agbayani, Jagdeep K. Sandhu, Bassel Akache, Charis McPherson, Lise Deschatelets, Renu Dudani, Melissa Hewitt, Yimei Jia, Lakshmi Krishnan, Michael J. McCluskie

**Affiliations:** Human Health Therapeutics, National Research Council of Canada, Montreal Rd. 1200, Ottawa, ON K1A 0R6, Canada; Felicity.Stark@nrc-cnrc.gc.ca (F.C.S.); Gerard.Agbayani@nrc-cnrc.gc.ca (G.A.); Jagdeep.Sandhu@nrc-cnrc.gc.ca (J.K.S.); Bassel.Akache@nrc-cnrc.gc.ca (B.A.); charismcpherson@cmail.carleton.ca (C.M.); Lise.Deschatelets@nrc-cnrc.gc.ca (L.D.); Renu.Dudani@nrc-cnrc.gc.ca (R.D.); Melissa.Hewitt@nrc-cnrc.gc.ca (M.H.); Yimei.Jia@nrc-cnrc.gc.ca (Y.J.); Lakshmi.Krishnan@nrc-cnrc.gc.ca (L.K.)

**Keywords:** cancer, immunotherapy, checkpoint, PD-1, CTLA-4, archaeal, archaeosomes, vaccine, adjuvant, melanoma, TIL, tumor infiltrating lymphocyte

## Abstract

Archaeosomes are liposomes composed of natural or synthetic archaeal lipids that when used as adjuvants induce strong long-lasting humoral and cell-mediated immune responses against entrapped antigens. However, traditional entrapped archaeosome formulations have only low entrapment efficiency, therefore we have developed a novel admixed formulation which offers many advantages, including reduced loss of antigen, consistency of batch-to-batch production as well as providing the option to formulate the vaccine immediately before use, which is beneficial for next generation cancer therapy platforms that include patient specific neo-antigens or for use with antigens that are less stable. Herein, we demonstrate that, when used in combination with anti-CTLA-4 and anti-PD-1 checkpoint therapy, this novel admixed archaeosome formulation, comprised of preformed sulfated lactosyl archaeol (SLA) archaeosomes admixed with OVA antigen (SLA–OVA (adm)), was as effective at inducing strong CD8^+^ T cell responses and protection from a B16-OVA melanoma tumor challenge as the traditionally formulated archaeosomes with encapsulated OVA protein. Furthermore, archaeosome vaccine formulations combined with anti-CTLA-4 and anti-PD-1 therapy, induced OVA-CD8^+^ T cells within the tumor and immunohistochemical analysis revealed the presence of CD8^+^ T cells associated with dying or dead tumor cells as well as within or around tumor blood vessels. Overall, archaeosomes constitute an attractive option for use with combinatorial checkpoint inhibitor cancer therapy platforms.

## 1. Introduction

With the arrival of checkpoint inhibitor-based immunotherapy, the once low five-year survival rates for many cancer types, including metastatic melanoma, are dramatically improving. In 2011, the breakthrough checkpoint inhibitor (CPI), cytotoxic T-lymphocyte-associated protein-4 antibody (anti-CTLA-4), ipilimumab, was approved for use with metastatic melanoma and is attributed with doubling one- and two-year survival rates [1]. In 2014, the program death receptor-1 antibody (anti-PD-1), pembrolizumab, was approved for use in advanced melanoma, and shortly after it was extended to be used as a first-line treatment for many cancer indications [2,3]. A combination of anti-CTLA-4 and anti-PD-1 targeting antibodies are now commonly used to treat unresectable and/or advanced melanoma with great success and an overall median survival rate that has not yet been reached [4]. Within this relatively short time window, the three-year survival rates for people diagnosed with metastatic melanoma has jumped from 4.7% to 63% [5,6]. Along with the many successes of CPIs in clinical trials, it has become clear that tumor-infiltrating antigen-specific effector CD8^+^ T cell responses are essential for successful CPI therapy, and that to further improve responses, there is a need for combinatorial treatments, such as vaccines, that can activate and mobilize tumor-specific CD8^+^ T cells.

Archaeosomes have been consistently proven to be a versatile adjuvant capable of inducing potent immunity in a number of animal models of disease. To date, archaeosomes have been used with multiple antigens, including listeriolysin (LLO), tyrosinase-related-protein-2 (Trp2), glycoprotein-100 (Gp100), Hepatitis-B surface antigen (HBsAg), influenza haemagglutinin (HA) and Hepatitis-C virus E1E2 heterodimer (HCV E1E2) [7,8,9,10,11,12], inducing both strong antibody responses as well as potent cellular responses that afford protection against infections and/or tumor challenge in murine models [7,8,12,13,14,15]. Archaeosomes are lipid vesicles composed of glycerolipids derived from archaea. Traditionally formulated archaeosomes are made with a heterogeneous mix of total polar lipids (TPL) derived from archaea such as *Methanobrevibacter smithii* (MS) and are used to deliver entrapped antigens [7,16,17]. We have previously shown that traditionally formulated MS liposomes with encapsulated ovalbumin (MS–OVA) induced OVA-CD8^+^ T cell responses and delayed the progression of the solid B16-OVA melanoma tumor [8,15]. An elevated expression of PD-1 was also detected on tumor infiltrating OVA-CD8^+^ T cells, which indicated that CD8^+^ T cell activity was suppressed in the tumor. However, this also provided an opportunity to use anti-PD-1 therapy to alleviate CD8^+^ T cell suppression and improve cytotoxic function and we demonstrated that the combination of MS–OVA (with OVA encapsulated) along with anti-PD-1, anti-PD-L1 and anti-CTLA-4 therapy could induce long-lasting protection from tumor growth [15].

Although traditionally formulated archaeosome vaccines are capable of efficient delivery of antigen and inducing strong humoral and cell-mediated immune responses, there are certain drawbacks associated with using TPL formulations, including difficulty in maintaining batch-to-batch consistency in the proportions of lipid species that are naturally expressed by growing archaea and a low antigen entrapment efficiency (typically 5–40%), resulting in increased production costs and variability in the finalized formulation. To overcome these difficulties, we have developed a simplified archaeosome lipid formulation using a single glycolipid composed of a sulfated saccharide group covalently linked to the free *sn-1* hydroxyl backbone of an archaeal core lipid (sulfated lactosyl archaeol, SLA), which when simply admixed with soluble antigen prior to immunization could induce comparable humoral and cellular immune responses to those induced by the traditional method of entrapping antigen within the archaeosome vesicle [10,18,19]. Advantages to this formulation include consistency of production, reduced costs and ease of synthesis while still retaining a similar level of adjuvanticity, as observed with conventional archaeosomes. In this study, we evaluate whether semi-synthetic SLA archaeosomes admixed with OVA and combined with CPI immunotherapy (anti-PD-1 and anti-CTLA-4) can induce protective immunity in a therapeutic solid melanoma tumor mouse model (B16-OVA).

## 2. Experimental Section

### 2.1. Mouse Strains

Six–eight-week-old female C57BL/6 were obtained from Charles River Laboratories (Senneville, QC, Canada). Mice were maintained at the small animal facility of the National Research Council Canada (NRC) in Ottawa, Canada, in accordance with the guidelines of the Canadian Council on Animal Care. All animal use protocols were approved by the NRC Animal Care Committee (2016.02: 2 March 2016).

### 2.2. Tumor Model (B16-OVA, B16 Melanoma)

B16 and B16F0-OVA (expressing plasmid derived full length OVA) cells were obtained from Dr. Edith Lord (University of Rochester, Rochester, NY, USA) and cultured as described previously [20,21]. Solid tumors were induced with s.c. injection of 5 × 10^5^ B16-OVA cells. From day 7 onwards, a detectable solid tumor was measured using Digimatic Digital calipers (Mitutoyo 500196–, Aurora, IL, USA). Tumor size, expressed in mm^2^, was obtained by multiplication of diametrically perpendicular measurements. Animals were monitored throughout the duration of the study. However, in order to minimize pain and discomfort, mice were humanely euthanized when the tumor exceeded 17 mm in any direction, was ulcerated (bleeding, leaking fluid or large cavity) or when the mice showed signs of clinical illness (ruffled fur, very little activity, hunched posture, eyes squeezed shut or very sickly).

### 2.3. Vaccine Preparation and Route of Immunization

Semi-synthetic archaeosome lipids were prepared by first purifying archaeol from *Halobacterium salinarum* (ATCC 33170, Manassas, VA, USA) and using it as a building block to attach a synthetic head-group, sulfated lactose. The resulting lipid was sulfated lactosyl archaeol (SLA; 6’-sulfate-β-D-Gal*p*-(1,4)-β-D-Glc*p*-(1,1)-archaeol) and was synthesized as described previously [22,23]. *M. smithii* (MS) total polar lipids were extracted as described previously [22,24,25]. SLA archaeosomes were formed and simply admixed with OVA, hereafter referred to as SLA–OVA (adm) as described previously [26]. Conventional SLA archaeosomes with entrapped ovalbumin, hereafter referred to as SLA–OVA (enc) were formed as described previously [26] and were diluted in phosphate-buffered saline (PBS; Thermo Fisher Scientific, Ottawa, ON, Canada) (20 µg OVA/100 µL PBS). As a comparator, conventional MS archaeosomes with entrapped ovalbumin, hereafter referred to as MS–OVA (enc) were also generated as previously described [15,24]. Mice received a subcutaneous (s.c.) injection of archaeosome-formulated OVA (20 µg OVA) at the base of the tail at various time-points after the tumor challenge (see Section 2.4 below). Numbers of animals per group and vaccination regimen are indicated in the figure legends.

### 2.4. Checkpoint Inhibitor Combination Therapy

C57BL/6 mice were given 5 × 10^5^ B16-OVA or B16 cells by s.c. injection in the dorsal flank; 3, 8 and 18 days later 20 μg MS–OVA (enc), SLA–OVA (enc) or SLA–OVA (adm) was injected s.c. at the base of the tail away from the tumor site. A total of 100 μg anti-CTLA-4, clone 9D9 (In Vivo Plus, BioXcell, West Lebanon, NH, USA) was given s.c. alongside the archaeosome treatment (days 3, 8 and 18). Where indicated, 250 μg of anti-PD-1, clone RMP1-14 (In Vivo Plus, BioXcell, West Lebanon, NH, USA) was given by intraperitoneal (i.p.) injection on days 9, 12, 15 and 18 (dosage and timing based on previously published data [15]).

### 2.5. Cellular Processing and Detection of OVA-Specific CD8^+^ T Cells

Tumor and spleen samples were collected at various time points during the study when the mice had reached their humane endpoint. Spleen and tumor samples were processed as described previously [15,27]. Briefly, spleen samples were mashed between two frosted glass slides to obtain a single-cell suspension. Tumor samples were cut with scissors into small pieces, digested for 1 h at 37 °C with 1 mg/mL collagenase type 4 (Worthington Biochemical Corporation, Lakewood, NJ), 0.1 mg/mL of hyaluronidase (Sigma-Aldrich, Oakville, ON, Canada) and 200 units of DNase (Roche Diagnostics, Indianopolis, IN, USA). Cells were washed in PBS and then layered over discontinuous gradients of 35% and 70% Percoll^®^ (GE Healthcare; Sigma-Aldrich, Oakville, ON, Canada) and centrifuged at 2000× *g*, for 20 min at 4 °C with the brakes off. Lymphocytes were isolated from the 35/70% gradient interphase. Single-cell suspensions were stained for flow cytometric analysis. First, Fc receptors were blocked with Fc receptor antibody, anti-CD16/CD32 (FcγRIII/II) for 5 min at 4 °C. Cells were stained with MHC tetramer H-2Kb OVA-BV421 (MBL international corporation, Woburn, MA, USA) for 20 min followed by staining for CD90.2-APC-eFluor^®^ 780 (eBioscience^TM^ ThermoFisher Scientific, Waltham, MA, USA), CD45-PerCP-Cy5.5 (Becton, Dickinson and Company, Franklin Lakes, NJ, USA), CD8-FITC (MBL International Corporation, Woburn, MA, USA), PD-1-BV786 (Becton, Dickinson and Company, Franklin Lakes, NJ, USA) and CTLA-4-CF594 (Becton, Dickinson and Company, Franklin Lakes, NJ, USA). Cells were washed with PBS and stained with the LIVE/DEAD™ Fixable Blue Dead Cell Stain Kit for UV excitation (ThermoFisher Scientific, Waltham, MA, USA), and then fixed with BD Cytofix^TM^ fixation buffer (Becton, Dickinson and Company, Franklin Lakes, NJ, USA) and acquired on a BD LSR Fortessa^TM^ analyzer (Becton, Dickinson and Company, Franklin Lakes, NJ, USA). Forward scatter-area (FSC-A) vs. side scatter-area (SSC-A) parameters were used to locate lymphocytes and exclude debris. Doublets were excluded according to their FSC-height (FSC-H) and FSC-A characteristics. Cells that were negative for the LIVE/DEAD™ Fixable dye were identified as live cells. CD45^+^, CD90.2^+^ CD8^+^ and OVA-tetramer^+^ cells were gated. Single-stained antibody controls were used to set compensation values. Fluorescence minus one (FMO) gating controls were used to distinguish background fluorescence from positive cell populations. Flow cytometry data were analyzed using FlowJo^®^ 10 (FlowJo LLC, Ashland, OR, USA).

### 2.6. Statistical Analysis

Data were analyzed using GraphPad Prism^®^ (GraphPad Software, San Diego, CA, USA). One-way and two-way analysis of variance (ANOVA) followed by post-hoc analysis using Tukey’s (comparison between all groups) multiple comparison tests were used as indicated in the figure legends. Outliers were identified with a ROUT co-efficient of 1%, however no outliers were identified. Survival curve analyses were carried out using the Gehan–Breslow Wilcoxon test, which best tests the early difference observed between treated and untreated test groups. For all analyses, differences were considered to be not significant with *p* > 0.05. Hazard ratio’s (HR’s) were calculated by determining the area under the survival curve (AUC) with a restricted endpoint of 70 days (rAUC) of a treatment group and dividing it by the rAUC of the untreated control group.

### 2.7. Processing and Immunohistochemical Detection of Tumor-Infiltrating Immune Cells

Mice were anesthetized with isoflurane and tumors were removed and fixed for 48 h at room temperature in formalin-free 1× zinc fixative solution prepared from a 10× stock (Becton, Dickinson and Company, Franklin Lakes, NJ, USA) and then transferred to 70% ethanol. Tumors were embedded in paraffin and 5 µm-thick serial sections were cut and dried overnight. Sections were stained with hematoxylin and eosin (H&E) and serial sections were subjected to immunohistochemistry (IHC) using the automated Bond-Max III (Leica Biosystems, Wetzlar, Germany). Immunostaining was carried out using a modified protocol J. Sections were incubated for 15 min at room temperature with 1:500 dilutions of rabbit monoclonal antibodies to CD45 and CD8 or 1:100 dilution of CD4 (Cell Signaling, Danvers, MA, USA) followed by incubations for 30 min with Polymer AP. After incubation for 15 min with a Bond Polymer Refine Red Detection system (Leica Biosystems, Wetzlar, Germany), slides were counterstained for 5 min with hematoxylin, dehydrated, cleared and mounted. Red precipitate was detected at the sites of antigen-antibody reaction. Zinc-fixed paraffin embedded mouse spleens from tumor bearing mice were used as positive controls. Each antibody was titrated and optimized for the demonstration of CD45, CD8 and CD4 immune infiltrate. Negative controls included omission of primary antibody and incubation with secondary antibody alone. All images were captured on an Olympus IX81 microscope equipped with a DP27 color CCD camera using the 20× or 60× oil objectives (Shinjuku, Tokyo, Japan).

### 2.8. Histological Scoring

Tumor sections immuno-stained with antibodies to CD45 and CD8 were evaluated in a blinded manner by two independent observers and assigned a histological score after examining 8–10 fields at 40× magnification for each tumor section on an Olympus CH30 bright field microscope (Shinjuku, Tokyo, Japan). Two to three tumor sections were scored, and a total of 2 to 4 individual mice from each group were evaluated for CD45^+^ cells and CD8^+^ cells. A four-tier grading system (score 0–3) based on the density (mild, moderate or marked infiltration) of immune infiltrate was set up based on a standardized methodology of assessing tumor-infiltrating lymphocytes in melanoma [28,29]. Immune infiltrate grades were defined as follows: grade 0, absence of immune cells; grade 1, mild (1–10 immune cells/field); grade 2, moderate (10–25 immune cells/field); and grade 3, marked (>25 immune cells/field). First the tumors were examined under low magnification in order to select for high-powered 40× fields to grade the immune infiltrate at the tumor margin and in the tumor nests; both stromal and intratumoral infiltrate was evaluated. Immune infiltrate outside of the tumor, i.e., in the capsule and neutrophils in the necrotic areas, were excluded.

## 3. Results

### 3.1. SLA–OVA (adm) Immunization Synergizes with Anti-CTLA-4 and Anti-PD-1 Therapy to Provide Protection in a B16-OVA Melanoma Model

**Figure 1:**
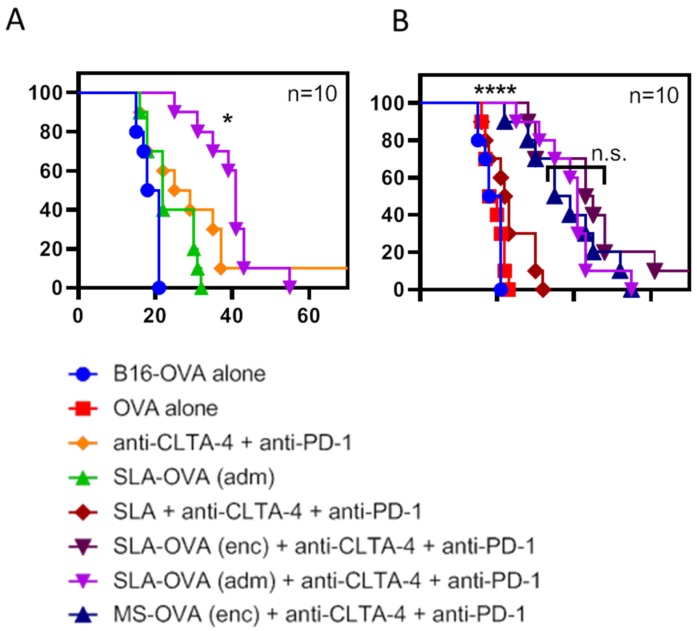
Survival of C57BL/6 mice challenged with a solid melanoma tumor and treated with archaeosomes in combination with anti-PD-1 and anti-CTLA-4. C57BL/6 mice were given 5 × 10^5^ B16-OVA tumor cells s.c. in the dorsal flank. Mice were treated with 20 μg of MS–OVA (enc), SLA–OVA (adm) or OVA alone by s.c. injection at the base of the tail away from the tumor site on days 3, 8 and 18. A total of 100 μg anti-CTLA-4 (Clone 9D9) was given s.c. alongside the archaeosome vaccination, and 250 μg of anti-PD-1 (RMP1-14) was given i.p. on day 9, 12, 15 and 18. Survival is plotted against time, and treatment groups are separated to better illustrate differences between groups. SLA–OVA (adm) therapy is compared to SLA–OVA (adm) combined with anti-PD-1 and anti-CTLA-4 therapy (**A**). SLA–OVA (adm), SLA–OVA (enc) or MS–OVA (enc) therapy in combination with anti-PD-1 and anti-CTLA-4 is compared (**B**). This survival assay was repeated once. Survival statistics were performed using the Gehan–Breslow-Wilcoxon test, * *p* < 0.05: SLA–OVA (adm) + anti-CTLA4 + anti-PD-1 > anti-CTLA4 + anti-PD-1; n.s. *p* > 0.1: SLA–OVA (adm) + anti-CTLA4 + anti-PD-1 = SLA–OVA (enc) + anti-CTLA4 + anti-PD-1 = MS–OVA (enc) + anti-CTLA4 + anti-PD-1; **** *p* < 0.0001: SLA–OVA (adm) + anti-CTLA4 + anti-PD-1 AND SLA–OVA (enc) + anti-CTLA4 + anti-PD-1 AND MS–OVA (enc) + anti-CTLA4 + anti-PD-1 > each control group shown on panel B.

**Figure 2:**
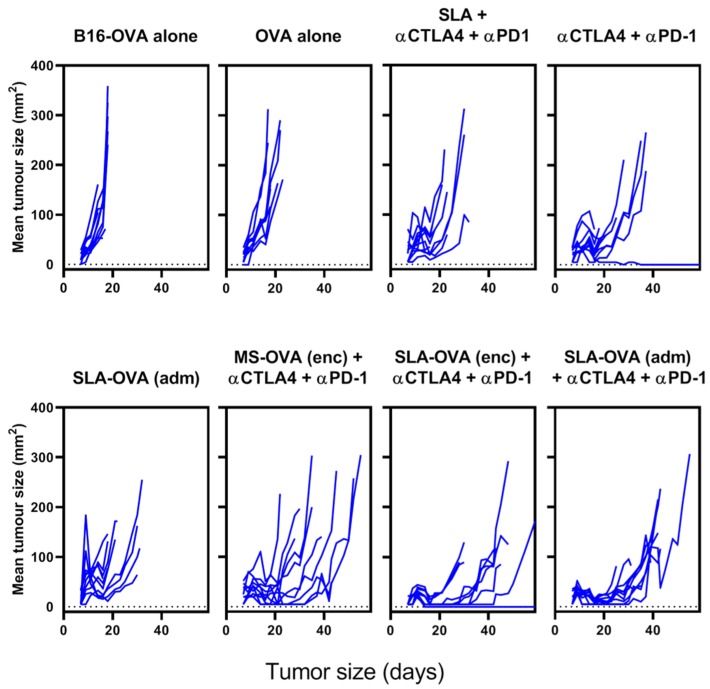
Solid tumor growth over time on C57BL/6 mice challenged with a solid melanoma tumor and treated archaeosomes in combination with anti-PD-1 and anti-CTLA-4. C57BL/6 mice were given 5 × 10^5^ B16-OVA tumor cells s.c. in the dorsal flank and treated as described in Figure 1. Perpendicular tumor measurements were taken, and tumor growth is plotted against time for each treatment group.

MS–OVA archaeosomes have been previously used to deliver entrapped antigen and induce anti-tumor CD8^+^ T cell responses [10,15,16,27]. Using the solid B16-OVA melanoma tumor model, we have previously demonstrated a synergy between MS–OVA (enc) and CPI therapy (anti-PD-1, anti-programmed death-ligand 1 (anti-PD-L1) and anti-CTLA-4) in promoting long-term tumor-free survival in C57BL/6 mice [15]. In this study, we evaluated whether our novel SLA archaeosome formulation in the form of empty archaeosomes simply admixed with ovalbumin, SLA–OVA (adm), could enhance survival in a B16-OVA melanoma model when used alone or in combination with CPIs. We compared these responses with those induced by archaeosome formulations with encapsulated antigens, namely MS-OVA (enc) and SLA–OVA (enc), in combination with CPIs. Non-vaccinated control mice reached the humane endpoint of 17 mm in any direction or tumor ulceration with a median survival of 19.5 days (Figure 1 and Figure S1), wh41ereas therapeutic SLA–OVA (adm) immunization extended the median survival to 22 days (*p* < 0.05, Figure 1). Survival could be further increased to 41 days if SLA–OVA (adm) was used in combination with anti-PD-1 and anti-CTLA-4 therapy (*p* < 0.05) with a median survival greater than that obtained with CPIs alone (Figure 1A). As would be expected, tumors were also smaller in these groups (Figure 2). Similar effects with these treatment regimens on survival were observed in a repeat experiment [30]. HRs were also calculated to determine the effect of treatment on overall survival by dividing the rAUC of a treatment group to the rAUC of the untreated control group. An HR of 1 would indicate no effect of the treatment compared to control. An HR of less than 0.50 would indicate a greater than 50% enhancement of survival. All three complete treatment groups tested had an HR value of less than 0.5, these include SLA–OVA (adm) + anti-CTLA-4 + anti-PD-1 (HR = 0.45), SLA–OVA (enc) + anti-CTLA-4 + anti-PD-1 (HR = 0.40), MS–OVA (enc) + anti-CTLA-4 + anti-PD-1 (HR = 0.46). Additionally, all three formulations gave equivalently high median survival rates with no significant differences observed (*p* > 0.1; 41, 44 and 37 days, respectively) (Figure 1B), while tumor growth appeared to be slightly slower with SLA–OVA compared to MS–OVA treatment (Figure 2).

### 3.2. SLA–OVA (adm) Therapy Increased the Frequencies of OVA-CD8^+^ T Cells in the Spleen and Tumor

Spleens and whole tumors were collected from B16-OVA mice at humane endpoints and processed for flow cytometric analysis of OVA-CD8^+^ T cells using H-2kb OVA tetramers (Figure S2A). Mice that received no treatment, OVA protein alone, anti-CTLA-4 + anti-PD-1 or SLA + anti-CTLA-4 + anti-PD-1 (no OVA protein) induced OVA-CD8^+^ T cells below the limit of detection (data not shown). In contrast, mice that received either SLA–OVA (adm) alone, or any of the archaeosome formulations, i.e., SLA–OVA (adm), SLA–OVA (enc), or MS–OVA (enc), in combination with CPIs had detectable OVA-CD8^+^ T cells in both the spleen and the tumor (Table 1), although no significant differences between groups was observed. The expression of CPI targets, CTLA-4 and PD-1, was measured on OVA-CD8^+^ T cells in the spleen and tumor of mice that reached their humane endpoint, however no significant differences between groups were observed (data not shown). We also evaluated whether the addition of a third CPI, anti-PD-L1, could further enhance protection against B16-OVA melanoma. However, overall survival and tumor growth was not significantly different compared to SLA–OVA (adm) + anti-PD-1 and anti-CTLA [30].

### 3.3. Histological Properties of B16 Tumors

Histological examination of H&E-stained B16 control and B16-OVA tumors revealed poorly differentiated tumors with considerable variation in cell and nuclear size (Figure S2A). Tumors were typically surrounded by a capsule with distinct regions of viable and non-viable (necrotic) tumor tissue (Figure S2B). Tumor cells were organized into clusters with clear stroma surrounding them (Figure S2A,D,E). Most tumors show neutrophil infiltration, which was present mainly at the tumor periphery (i.e., capsule, Supplemental Figure S2A) and in necrotic areas (Figure S2B,C). Based on our examination of three B16 control and four B16-OVA tumors, it is clear that they all had necrotic areas with neutrophils as the predominant infiltrating immune cell type. No lymphocyte infiltration was seen in B16-OVA tumors (from untreated mice), thus representing a good model system to test immunotherapeutic strategies. B16-OVA tumors treated with checkpoint inhibitors (Figure S2D) or checkpoint inhibitors and SLA–OVA (Figure S2E) had increased lymphocyte infiltration and tumor cell death (dark pigmented cells, Figure S2D,E).

### 3.4. Immunohistochemical Evaluation of CD8^+^ T Cells in B16-OVA Tumor-Bearing Mice Receiving SLA–OVA and Checkpoint Therapy

**Figure 3:**
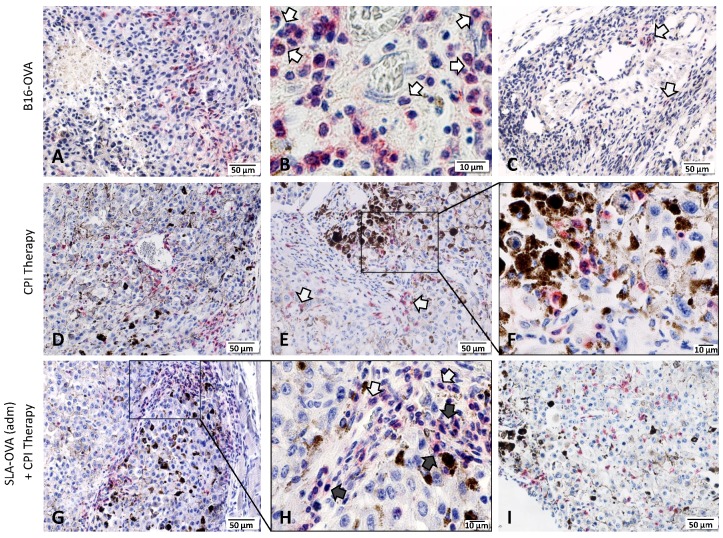
Immunohistochemical demonstration of immune-infiltrating cells in B16-OVA tumor-bearing mice. Tumors were collected at the humane endpoint and were fixed in zinc fixative and immunostained with rabbit monoclonal antibodies against CD45, CD4 and CD8, developed using a Bond Polymer Refine Red Detection system (red) and counterstained with hematoxylin (blue). Top panel: Photomicrographs of a B16-OVA tumor showing the presence of CD45^+^ cells around necrotic area (**A**), morphologically resembling neutrophils (**B**) and few CD4^+^ T cells at the tumor margin (**C**). Middle panel: Photomicrographs of a B16-OVA tumor treated with CPI therapy showing CD45^+^ cells in the tumor stroma (**D**) and CD8^+^ cells in the viable (arrows) and non-viable tumor tissue (**E**). A black box in panel E was magnified to show closely associated CD8^+^ cells with dead/dying tumor cells (**F**). Bottom panel: Photomicrographs of a B16-OVA tumor treated with a combination of CPI therapy and SLA–OVA, showing CD45^+^ cells in the tumor stroma (**G**,**H**), morphologically resembling neutrophils (**H**) and numerous intra-tumoral CD8^+^ cells in the viable tumor tissue (**I**). A black box in panel G is magnified to show neutrophil morphology in panel H.

**Figure 4:**
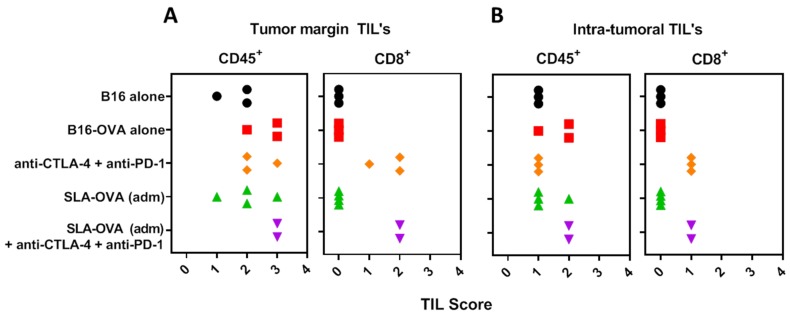
Semi-quantitative analysis of the immune cell infiltrate. Using a four-tier scoring system (0, 1, 2 and 3), CD45^+^ and CD8^+^ cells were semi-quantified in the tumor margin (**A**) and within the tumor (**B**). A score of “0” indicates no cells in the field, “1” indicates mild infiltrate of 1–10 immune cells/field, “2” indicates moderate infiltration of 10–25 immune cells/field and a score of “3” indicates marked infiltration with >25 cells/field. 40× fields were used to grade the immune cell infiltrates. Necrotic areas were excluded from analysis. Groups are color-matched to the survival curves in Figure 1.

Increased tumor infiltrating lymphocytes and their location in the tumor is a key prognostic indicator in melanoma [30]; therefore, we investigated the extent of immune cell infiltration by IHC (Figure 3). Non-immunized B16-OVA tumor-bearing mice had extensive CD45^+^ staining and these cells were present in the capsule and tumor margin, viable tumor tissue and in necrotic areas (Figure 3A). Under higher magnification, CD45^+^ cells resembled typical polymorphonuclear cells, suggestive of neutrophil infiltration (Figure 3B). Few CD4^+^ T cells were encountered at the tumor margin (Figure 3C). No CD8^+^ T cells were seen at the tumor margin or within tumor nests (Figure 4). Treatment of B16-OVA tumor-bearing mice with checkpoint inhibitors recruited a large number of CD45^+^ cells (Figure 3D), including many CD8^+^ cells (Figure 3E). The majority of the CD8^+^ cells were present at the tumor margin with appreciable numbers in the tumor stroma and tumor vasculature, as well as penetrating the viable and non-viable tumor tissue (Figure 3E). A representative high magnification example shows that CD8^+^ cells are close to the viable and non-viable tumor cells (Figure 3F). B16-OVA tumor-bearing mice treated with checkpoint inhibitors and SLA–OVA had the highest numbers of CD45^+^ cells (Figure 3G) and CD8^+^ cells and these cells were found close to dying or dead tumor cells (Figure 3H) and were widely dispersed throughout the viable tumor tissue (Figure 3I). Tumors from mice treated with checkpoint inhibitors and OVA also had an increased number of CD8^+^ cells (Figure 4). In contrast, tumor-bearing mice receiving SLA–OVA alone had CD4^+^ T cells at the tumor margin but very few CD8^+^ T cells. Interestingly, tumor-bearing mice that received a combination therapy of checkpoint inhibitors and SLA–OVA or OVA were associated with increased tumor cell death (Figure 3G,H). Dead tumor cells had increased expression of melanin and appeared as darkly pigmented cells (Figure 3G,H). The results of the photomicrographs were corroborated with semi-quantitative analysis of the immune cell infiltrate in the tumor margin (Figure 4A) and intratumorally (Figure 4B). Using the four-tier scoring system [28,29], we found that the grade for tumor infiltrating CD8^+^ cells for B16-OVA tumor-bearing mice treated with CPI’s alone or in combination with SLA–OVA (adm) was 1 to 2 at the tumor margin and 0 to 1 in tumor nests; conversely, B16-OVA tumor-bearing mice injected with the vehicle alone had a grade of 0. Overall, CPI treated mice had an increased infiltration of CD8^+^ immune cells at the tumor margin and within tumor nests.

## 4. Discussion

The use of CPI therapy is considered a breakthrough in the field of cancer therapy, but their success has been limited to patients with existing tumor-specific T cell responses. Patients with T cell-inflamed tumors, also known as “hot” tumors are often found to have suppressed T cell cytotoxicity, but this can be reversed with a CPI treatment regime. However, “cold” tumors, defined as having a poor T cell infiltration either due to physical tumor barriers (extracellular matrix) that exclude T cells or due to a general absence of T cell responses in the patient, are often found to be not responsive to CPI based immunotherapy [31]. Unlike human melanomas that develop slowly and with significant T cell infiltrates, the B16-OVA melanoma mouse tumor is a good model of a cold tumor system as the tumor develops quickly (7–9 days) without significant T cell infiltration. Despite these differences, human and B16-OVA melanoma tumors share high susceptibility to killing by antigen-specific CD8^+^ T cells. These “cold” tumor systems have been the focus of intense research of late in an effort to find combinatorial treatments to enhance T cell activation as well as their proliferation and infiltration into tumors [32,33]. Indeed, recent and active clinical trials for melanoma are combining the now standard of care CPI therapy with treatments such as autologous tumor cell therapy, including Dorgenmeltucel-L or VIGIL-^TM^, mRNA mutanome therapy (RO7198457) and a number of patient-specific peptide vaccines, such as NEO-PV-01, VB10.NEO, 6MHP and GEN-009, some of which are being combined with adjuvants such as Poly-ICLC (Hiltonol) or Montanide ISA-51 [34,35,36,37,38,39,40,41]. The combination of an adjuvant, e.g., Poly-ICLC (Hiltonol) or CpG with CPIs has also been evaluated using in situ vaccination models [42,43]. Therefore, while CPI therapy has marked a clear turning point for cancer therapy as a whole, adjuvants with a proven record of stimulating tumor-specific CD8^+^ T cells are well-poised to join the battle [32].

Archaeal lipid adjuvants are a good potential candidate for use in combination with CPI’s, as evidenced by their proven safety and efficacy in mice [19,44]. In multiple pre-clinical studies, they have been shown to activate antigen-specific CD8^+^ T cell responses [15,19,26,27] and generate protective immunity in tumor models, e.g., B16-OVA melanoma [7,13,19,23,26,27], and against multiple infectious diseases, e.g., H1N1 influenza [11], *Listeria monocytogenes* [7,12], *Trypanosoma cruzi* [13] and *Mycobacterium tuberculosis* [14]. When compared to commonly used commercial adjuvants, such as Poly(I:C) or Montanide, archaeal lipid vaccines were found to elicit equal or greater antigen specific CD8^+^ T cell-mediated cytotoxicity and IgG responses [10]. SLA-based archaeosomes can also induce local cytokine production at the site of injection even in the absence of an antigen [19]. While cytokine production was not measured in this study, the use of empty SLA archaeosomes combined with anti-PD-1 and anti-CTLA-4 therapy (without OVA antigen) did not cause any increase in median survival compared to anti-PD-1 and anti-CTLA-4 alone (22.5 vs. 27 days, respectively).

In this study, SLA archaeosome adjuvanted OVA formulations synergized with dual CPI therapy to improve median survival in the murine melanoma model, as was previously observed with the traditionally formulated *M. smithii* archaeosomes with OVA encapsulated. Similarly, we have observed a synergy of archaeosomes with single checkpoint inhibitors (anti-CTLA-4 or anti-PD-1) but with a shortened median survival [15]. While the use of single checkpoint inhibitors (anti-CTLA-4 or anti-PD-1) in human cancer therapies has induced remarkable long-term protective responses, these were often limited to a minority of patients [45,46,47]; investigators were thus determined to find beneficial combinatorial treatments to improve responses. Anti-CTLA-4 and anti-PD-1 combinations have been very successful in the clinic as evidenced in the CheckMate trials by the Bristol–Myers–Squibb Company. Most recently the five-year results from the Phase 3 CheckMate 067 clinical trial for metastatic melanoma showed a five-year overall survival rate of 52% for patients that received dual anti-CTLA-4 and anti-PD-1 therapy compared to 26% and 44% (respectively) for single CPI therapy [48]. Pre-clinical studies have also revealed that the enhanced protection of combination anti-CTLA-4 and anti-PD-1 therapy is not simply an additive effect but rather a distinct shift in CD8^+^ T cell phenotype and an increase in Th-1 CD4^+^ effector T cells [49]; this further supports the notion that these are not redundant pathways and a clear and distinct benefit is attained with their combination.

While it has been reported that anti-PD-1 and anti-CTLA-4 combination therapy increases the incidence and severity of adverse effects, these were observed to be linked to the beneficial anti-tumor immune response; an inherently anti-“self” immune response capable of targeting non-tumor “self” targets throughout the body [50]. Management of immune-related adverse events (irAEs) are often handled with corticosteroids or discontinuation of treatment and are often considered by clinicians to be manageable with proper dosing. In the mouse model, we did not observe irAEs during treatment; however, this complication was not expected as the duration of the study (from tumor injection to humane euthanasia) was much shorter compared to natural tumor development in a human (weeks vs. months/years) and anti-“self” immune responses would likely need more time to develop. As a next step, immunization with tumor associated antigens (known or neo-antigens) instead of OVA would add to the stringency of the model, it would also allow for the evaluation of the ability of the treatment regimen to overcome the important hurdle of breaking immunological tolerance. Since archaeosomes have previously been shown to break tolerance to the melanoma Trp2 protein, a follow-up study investigating CPI therapy in combination with SLA-Trp2 admix therapy would be a logical next step.

With a goal to advance archaeosome vaccine formulations and improve consistency of formulation and ease of use, the archaeosome lipid composition has evolved from being composed of total polar lipids derived from *M. smithii* to a single glycolipid, SLA. We have shown, in previous studies, that both lipid formulations are capable of inducing comparable immune responses to numerous antigens [11,18,23]. Most recently, we compared different archaeosome vaccine formulations and discovered that admixing antigen and archaeosomes induced similar or superior immune responses to an encapsulated formulation [26]. In a prophylactic vaccination influenza challenge model, we compared formulations that were composed of empty archaeosomes admixed with antigen with the conventional antigen entrapped archaeosomes and again observed comparable immune responses as well as similar protection from the influenza challenge [11]. While it was initially thought that entrapment of antigen within the archaeosome vesicle would enhance cytotoxic CD8^+^ T cell responses by delivering the antigen to the cytosol, allowing it to readily access MHC class I processing machinery that is necessary to initiate CD8^+^ T cell responses, these findings indicate that the inherent adjuvant effect of archaeosomes is sufficient to initiate CD8^+^ T cell responses towards exogenous antigens. Notably, our findings that an SLA admix archaeosome formulation functions similarly to an entrapped antigen archaeosome formulation are not out of place in the current vaccine adjuvant landscape as many other adjuvants, such as CpG or Poly (I:C), are capable of initiating CD8^+^ T cell responses towards exogenous antigen [10].

In this study when we compared different archaeosomes formulations, namely SLA–OVA (adm), SLA–OVA (enc) and MS–OVA (enc), in combination with CPIs we found that all three formulations increased survival, slowed tumor growth and increased the frequency of OVA-CD8^+^ T cells in both spleen and tumor compared to CPIs alone. Furthermore, when tumors of SLA–OVA and CPI-treated mice were collected at humane endpoints and assessed with immunohistochemistry, the majority of the tumor mass was found to be composed of dead cells, in contrast to control tumors that were largely viable (unpublished observations), suggesting that tumor viability may be a good surrogate marker for survival in this model. Interestingly, in a previous study, when we evaluated MS–OVA (enc) in combination with anti-PD-1, anti-CTLA-4 and anti-PD-L1 therapy, we were able to protect 70% of mice beyond 100 days after the tumor challenge [15], longer than obtained in the current study. However, this could be due to batch-to-batch variations in the proportion of lipid species in MS archaeosomes formulations. Indeed, this highlights one of the key benefits of our newer lipid formulation, which is well defined as a high purity single lipid, namely SLA. It is also possible that changing the source of mice (Jackson Laboratories vs. Charles River Laboratories) could explain the change in overall survival of the mice, as other groups have shown that differences in gut microbiome between sources of C57BL/6 has been linked to a difference in the growth kinetics and severity of B16 melanoma tumor progression with anti-PD-L1 therapy [51] or anti-CTLA-4 therapy [52], suggesting that the gut microbiome may one day be used as a predictor of responsiveness to immune checkpoint inhibitors [53].

Herein, we demonstrated that the combination of an archaeosome adjuvanted vaccine with two checkpoint inhibitors (anti-CTLA-4 and anti-PD-1) could enhance survival and reduce tumor growth compared to either treatment alone. All three vaccine formulations, SLA–OVA (adm), SLA–OVA (enc) and MS–OVA (enc) in combination with the checkpoint inhibitors anti-PD-1 and anti-CTLA-4 induced OVA-CD8^+^ T cells detectable in the spleen and tumor. Similarly, tumors assessed by IHC showed the presence of CD8^+^ T cells and they were found to be dispersed throughout both viable and non-viable tumor tissue. CD8^+^ T cells were found to be associated with tumor cells with increased expression of melanin as well as within or around tumor blood vessels. The SLA–OVA (adm) alone immunized mice assessed by IHC had CD45^+^ immune cell infiltrate but very few were CD8^+^ T cells. Interestingly, when tumors were assessed by IHC, it was found that mice therapeutically treated with any vaccine containing CPIs (anti-PD-1, anti-CTLA-4 and anti-PD-L1) harbored tumors that were largely non-viable compared to control mice or mice treated with SLA–OVA (adm) alone where the tumor tissue was mostly viable. Thus, it is possible that in the different treatment groups, mice could have succumbed to tumor-related illness for disparate reasons, either because the tumor reached a terminally large size and/or metastasized or because the tumor was dying. Rapid tumor cell death resulting in the release of tumor cells contents into the bloodstream is a life-threatening adverse effect, known as tumor lysis syndrome, associated with severe metabolic abnormalities, which has recently been associated with CPI therapy in some cancer patients [54,55]. The immune components necessary for clearing a dead or dying tumor were not investigated in this study but would be interesting to pursue in this particular tumor model.

Antigen-specific tumor-infiltrating CD8^+^ T cells are needed for successful cancer immunotherapy and are considered a relevant measure of cancer vaccine efficacy [56]. As such the development of adjuvants for use in cancer therapy is an important next step for combinatorial cancer therapy platforms. The latest archaeosome formulation (SLA), which is composed of empty semi-synthetic archaeosomes admixed with antigen cargo, offers many advantages over traditionally formulated encapsulated archaeosomes. The ability to mix the vaccine immediately before use is particularly useful for neo-antigen formulations; where batch-to-batch consistency, speed and ease of production are priorities for safety and efficacy in patient-specific therapies. Additionally, admixed formulations have minimal antigen loss during formulation, which is ideal as modern cancer antigens are often complex and expensive. We have shown that the latest generation of archaeal vaccine adjuvants, the synthetic SLA liposome admixed with antigen synergizes with anti-PD-1 and anti-CTLA-4 therapy to induce CD8^+^ T cells in the tumor and enhances protection from tumor development to a similar extent than the traditionally formulated MS liposomes with encapsulated antigen. Thus, archaeosomes are a promising addition to the cancer immunotherapy armamentarium.

## Figures and Tables

**Table 1:** Comparison of frequency of OVA-CD8^+^ T cells in the spleen and tumor of mice described in Figure 1.

Frequency of OVA-CD8^+^ T Cells of All CD8^+^ T Cells in the Spleen and Tumor at the Humane Endpoint				
	Spleen		Tumour	
	Geometric Mean	%95 CI (Lower + Upper)	Geometric Mean	%95 CI (Lower + Upper)
SLA-OVA (adm)	1.13	0.38 and 1.79	0.55	0.23 and 1.35
Anti- CTLA4 + anti-PD-1	0.17	−0.03 and 0.56	0.56	0.17 and 3.12
SLA-OVA (adm) + anti-CTLA4 + anti-PD-1	0.27	0.14 and 1.38	1.85	0.18 and 5.50
SLA-OVA (enc) + anti-CTLA4 + anti-PD-1	1.49	0.44 and 3.07	8.02	0.41 and 21.97
MS-OVA (enc) + anti-CTLA4 + anti-PD-1	1.27	0.55 and 1.37	1.74	0.67 and 3.04

Control groups not included above did not have detectable OVA-CD8 T cells. Outliers were removed with a ROUT coefficient Q of 1%, *n* = 5–7/gp.

## Supplementary Materials

Supplementary materials can be found at https://www.mdpi.com/2227-9059/7/4/91/s1.

## References

[1] Alexander W. (2016). The Checkpoint Immunotherapy Revolution: what started as a trickle has become a flood, despite some daunting adverse effects; new drugs, indications, and combinations continue to emerge. Pharm. Ther..

[2] Barone A., Hazarika M., Theoret M.R., Mishra-Kalyani P., Chen H., He K., Sridhara R., Subramaniam S., Pfuma E., Wang Y. (2017). FDA Approval Summary: Pembrolizumab for the Treatment of Patients with Unresectable or Metastatic Melanoma. Clin. Cancer Res..

[3] Pai-Scherf L., Blumenthal G.M., Li H., Subramaniam S., Mishra-Kalyani P.S., He K., Zhao H., Yu J., Paciga M., Goldberg K.B. (2017). FDA Approval Summary: Pembrolizumab for Treatment of Metastatic Non-Small Cell Lung Cancer: First-Line Therapy and Beyond. Oncologist.

[4] Lugowska I., Teterycz P., Rutkowski P. (2018). Immunotherapy of melanoma. Contemp. Oncol..

[5] Song X., Zhao Z., Barber B., Farr A.M., Ivanov B., Novich M. (2015). Overall survival in patients with metastatic melanoma. Curr. Med. Res. Opin..

[6] Callahan M.K., Kluger H., Postow M.A., Segal N.H., Lesokhin A., Atkins M.B., Kirkwood J.M., Krishnan S., Bhore R., Horak C. (2017). Nivolumab Plus Ipilimumab in Patients with Advanced Melanoma: Updated Survival, Response, and Safety Data in a Phase I Dose-Escalation Study. J. Clin. Oncol..

[7] Conlan J.W., Krishnan L., Willick G.E., Patel G.B., Sprott G.D. (2001). Immunization of mice with lipopeptide antigens encapsulated in novel liposomes prepared from the polar lipids of various Archaeobacteria elicits rapid and prolonged specific protective immunity against infection with the facultative intracellular pathogen, Listeria monocytogenes. Vaccine.

[8] Krishnan L., Deschatelets L., Stark F.C., Gurnani K., Sprott G.D. (2010). Archaeosome adjuvant overcomes tolerance to tumor-associated melanoma antigens inducing protective CD8 T cell responses. Clin. Dev. Immunol..

[9] Landi A., Law J., Hockman D., Logan M., Crawford K., Chen C., Kundu J., Ebensen T., Guzman C.A., Deschatelets L. (2017). Superior immunogenicity of HCV envelope glycoproteins when adjuvanted with cyclic-di-AMP, a STING activator or archaeosomes. Vaccine.

[10] Akache B., Stark F.C., Jia Y., Deschatelets L., Dudani R., Harrison B.A., Agbayani G., Williams D., Jamshidi M.P., Krishnan L. (2018). Sulfated archaeol glycolipids: Comparison with other immunological adjuvants in mice. PLoS ONE.

[11] Stark F.C., Akache B., Ponce A., Dudani R., Deschatelets L., Jia Y., Sauvageau J., Williams D., Jamshidi M.P., Agbayani G. (2019). Archaeal glycolipid adjuvanted vaccines induce strong influenza-specific immune responses through direct immunization in young and aged mice or through passive maternal immunization. Vaccine.

[12] Ansari M.A., Zubair S., Tufail S., Ahmad E., Khan M.R., Quadri Z., Owais M. (2012). Ether lipid vesicle-based antigens impart protection against experimental listeriosis. Int. J. Nanomed..

[13] Higa L.H., Corral R.S., Morilla M.J., Romero E.L., Petray P.B. (2013). Archaeosomes display immunoadjuvant potential for a vaccine against Chagas disease. Hum. Vaccin Immunother..

[14] Ansari M.A., Zubair S., Mahmood A., Gupta P., Khan A.A., Gupta U.D., Arora A., Owais M. (2011). RD antigen based nanovaccine imparts long term protection by inducing memory response against experimental murine tuberculosis. PLoS ONE.

[15] Stark F.C., Weeratna R.D., Deschatelets L., Gurnani K., Dudani R., McCluskie M.J., Krishnan L. (2017). An Archaeosome-Adjuvanted Vaccine and Checkpoint Inhibitor Therapy Combination Significantly Enhances Protection from Murine Melanoma. Vaccines.

[16] Krishnan L., Dicaire C.J., Patel G.B., Sprott G.D. (2000). Archaeosome vaccine adjuvants induce strong humoral, cell-mediated, and memory responses: Comparison to conventional liposomes and alum. Infect. Immun..

[17] Sprott G.D., Patel G.B., Krishnan L. (2003). Archaeobacterial ether lipid liposomes as vaccine adjuvants. Meth. Enzymol.

[18] McCluskie M.J., Deschatelets L., Krishnan L. (2017). Sulfated archaeal glycolipid archaeosomes as a safe and effective vaccine adjuvant for induction of cell-mediated immunity. Hum. Vaccin. Immunother..

[19] Akache B., Stark F.C., Iqbal U., Chen W., Jia Y., Krishnan L., McCluskie M.J. (2018). Safety and biodistribution of sulfated archaeal glycolipid archaeosomes as vaccine adjuvants. Hum. Vaccin. Immunother..

[20] Brown D.M., Fisher T.L., Wei C., Frelinger J.G., Lord E.M. (2001). Tumours can act as adjuvants for humoral immunity. Immunology.

[21] Dudani R., Chapdelaine Y., van Faassen H., Smith D.K., Shen H., Krishnan L., Sad S. (2002). Multiple mechanisms compensate to enhance tumor-protective CD8^+^ T cell response in the long-term despite poor CD8(+) T cell priming initially: Comparison between an acute versus a chronic intracellular bacterium expressing a model antigen. J. Immunol..

[22] Whitfield D.M., Yu S.H., Dicaire C.J., Sprott G.D. (2010). Development of new glycosylation methodologies for the synthesis of archaeal-derived glycolipid adjuvants. Carbohydr. Res..

[23] Whitfield D.M., Sprott D.G., Krishnan L. (2016). Sulfated-Glycolipids as Adjuvants for Vaccines.

[24] Krishnan L., Gurnani K., Dicaire C.J., van Faassen H., Zafer A., Kirschning C.J., Sad S., Sprott G.D. (2007). Rapid clonal expansion and prolonged maintenance of memory CD8^+^ T cells of the effector (CD44^high^CD62L^low^) and central (CD44^high^CD62L^high^) phenotype by an archaeosome adjuvant independent of TLR2. J. Immunol..

[25] Sprott G.D., Yeung A., Dicaire C.J., Yu S.H., Whitfield D.M. (2012). Synthetic archaeosome vaccines containing triglycosylarchaeols can provide additive and long-lasting immune responses that are enhanced by archaetidylserine. Archaea.

[26] Jia Y., Akache B., Deschatelets L., Qian H., Dudani R., Harrison B.A., Stark F.C., Chandan V., Jamshidi M.P., Krishnan L. (2019). A comparison of the immune responses induced by antigens in three different archaeosome-based vaccine formulations. Int. J. Pharm..

[27] Stark F.C., McCluskie M.J., Krishnan L. (2016). Homologous Prime-Boost Vaccination with OVA Entrapped in Self-Adjuvanting Archaeosomes Induces High Numbers of OVA-Specific CD8^+^ T Cells that Protect Against Subcutaneous B16-OVA Melanoma. Vaccines.

[28] Azimi F., Scolyer R.A., Rumcheva P., Moncrieff M., Murali R., McCarthy S.W., Saw R.P., Thompson J.F. (2012). Tumor-infiltrating lymphocyte grade is an independent predictor of sentinel lymph node status and survival in patients with cutaneous melanoma. J. Clin. Oncol..

[29] Hendry S., Salgado R., Gevaert T., Russell P.A., John T., Thapa B., Christie M., van de Vijver K., Estrada M.V., Gonzalez-Ericsson P.I. (2017). Assessing Tumor-Infiltrating Lymphocytes in Solid Tumors: A Practical Review for Pathologists and Proposal for a Standardized Method from the International Immuno-Oncology Biomarkers Working Group: Part 2: TILs in Melanoma, Gastrointestinal Tract Carcinomas, Non-Small Cell Lung Carcinoma and Mesothelioma, Endometrial and Ovarian Carcinomas, Squamous Cell Carcinoma of the Head and Neck, Genitourinary Carcinomas, and Primary Brain Tumors. Adv. Anat. Pathol..

[30] Stark F., McCluskie M. (2019). National Research Council of Canada, Ottawa, ON. In a repeat experiment, all treatment groups were repeated with the addition of a separate group that included anti-PD-L1 therapy. The addition of anti-PD-L1 did not further enhance survival in combination therapy with anti-PD-1, anti-CTLA-4 and the adjuvant SLA admixed with ovalbumin protein in a therapeutic B16-OVA melanoma model.

[31] Halse H., Colebatch A.J., Petrone P., Henderson M.A., Mills J.K., Snow H., Westwood J.A., Sandhu S., Raleigh J.M., Behren A. (2018). Multiplex immunohistochemistry accurately defines the immune context of metastatic melanoma. Sci. Rep..

[32] Chen D.S., Mellman I. (2017). Elements of cancer immunity and the cancer–immune set point. Nature.

[33] Kyi C., Postow M.A. (2016). Immune checkpoint inhibitor combinations in solid tumors: Opportunities and challenges. Immunotherapy.

[34] Mariathasan S., Turley S.J., Nickles D., Castiglioni A., Yuen K., Wang Y., Kadel E.E., Koeppen H., Astarita J.L., Cubas R. (2018). TGFβ attenuates tumour response to PD-L1 blockade by contributing to exclusion of T cells. Nature.

[35] Immunotherapy Study for Patients with Stage IV Melanoma. https://clinicaltrials.gov/ct2/show/NCT02054520.

[36] Pilot Study of VigilTM + Pembrolizumab for Advanced Melanoma. https://clinicaltrials.gov/ct2/show/NCT02574533.

[37] A Study of RO7198457 as a Single Agent and in Combination with Atezolizumab in Participants with Locally Advanced or Metastatic Tumors. https://clinicaltrials.gov/ct2/show/NCT03289962.

[38] A Personal Cancer Vaccine (NEO-PV-01) and APX005M or Ipilimumab with Nivolumab in Patients with Advanced Melanoma. https://clinicaltrials.gov/ct2/show/NCT03597282.

[39] A Personal Cancer Vaccine (NEO-PV-01) w/ Nivolumab for Patients with Melanoma, Lung Cancer or Bladder Cancer. https://clinicaltrials.gov/ct2/show/NCT02897765.

[40] A Study to Evaluate Safety, Feasibility, Efficacy of Multiple Dosing with VB10.NEO Immunotherapy in Patients with Locally Advanced or Metastatic Cancer. https://clinicaltrials.gov/ct2/show/NCT03548467.

[41] A Phase I/II Trial to Evaluate a Peptide Vaccine plus Ipilimumab in Patients with Melanoma. https://clinicaltrials.gov/ct2/show/NCT02385669.

[42] Safety, Tolerability, Immunogenicity, and Antitumor Activity of GEN-009 Adjuvanted Vaccine. https://clinicaltrials.gov/ct2/show/NCT03633110.

[43] A Phase 1/2 Study of in Situ Vaccination with Tremelimumab and IV Durvalumab plus PolyICLC in Subjects with Advanced, Measurable, Biopsy-Accessible Cancers. https://clinicaltrials.gov/ct2/show/NCT02643303.

[44] Hu-Lieskovan S., Ribas A. (2017). New combination strategies using PD-1/ L1 checkpoint inhibitors as a backbone. Cancer J..

[45] Haq K., Jia Y., Krishnan L. (2016). Archaeal lipid vaccine adjuvants for induction of cell-mediated immunity. Expert Rev. Vaccines.

[46] Pardoll D.M. (2012). The blockade of immune checkpoints in cancer immunotherapy. Nat. Rev. Cancer.

[47] Sharma P., Allison J.P. (2015). The future of immune checkpoint therapy. Science.

[48] Topalian S.L., Drake C.G., Pardoll D.M. (2015). Immune Checkpoint Blockade: A Common Denominator Approach to Cancer Therapy. Cancer Cell.

[49] Five-Year Outcomes for Opdivo (nivolumab) in Combination with Yervoy (ipilimumab) Demonstrate Durable Long-Term Survival Benefits in Patients with Advanced Melanoma | BMS Newsroom. https://news.bms.com/press-release/corporatefinancial-news/five-year-outcomes-opdivo-nivolumab-combination-yervoy-ipilimu.

[50] Wei S.C., Anang N.-A.A.S., Sharma R., Andrews M.C., Reuben A., Levine J.H., Cogdill A.P., Mancuso J.J., Wargo J.A., Pe’er D. (2019). Combination anti-CTLA-4 plus anti-PD-1 checkpoint blockade utilizes cellular mechanisms partially distinct from monotherapies. Proc. Natl. Acad. Sci. USA.

[51] Boutros C., Tarhini A., Routier E., Lambotte O., Ladurie F.L., Carbonnel F., Izzeddine H., Marabelle A., Champiat S., Berdelou A. (2016). Safety profiles of anti-CTLA-4 and anti-PD-1 antibodies alone and in combination. Nat. Rev. Clin. Oncol..

[52] Sivan A., Corrales L., Hubert N., Williams J.B., Aquino-Michaels K., Earley Z.M., Benyamin F.W., Lei Y.M., Jabri B., Alegre M.-L. (2015). Commensal Bifidobacterium promotes antitumor immunity and facilitates anti–PD-L1 efficacy. Science.

[53] Vétizou M., Pitt J.M., Daillère R., Lepage P., Waldschmitt N., Flament C., Rusakiewicz S., Routy B., Roberti M.P., Duong C.P.M. (2015). Anticancer immunotherapy by CTLA-4 blockade relies on the gut microbiota. Science.

[54] Gong J., Chehrazi-Raffle A., Placencio-Hickok V., Guan M., Hendifar A., Salgia R. (2019). The gut microbiome and response to immune checkpoint inhibitors: Preclinical and clinical strategies. Clin. Transl. Med..

[55] Brunnhoelzl D., Wang J. (2017). Acute tumor lysis syndrome after anti-pd-1 immunotherapy nivolumab for metastatic melanoma. J. Mol. Oncol. Res..

[56] Brunnhoelzl D., Weed M., Trepet R., Wang J. (2017). Tumor Lysis Syndrome Following a Single Atezolizumab Infusion for Metastatic Urothelial Carcinoma Involving Both Upper and Lower Tract. Arch. Can. Res..

