# Supplementary material for: Simplified Admix Archaeal Glycolipid Adjuvanted Vaccine and Checkpoint Inhibitor Therapy Combination Enhances Protection from Murine Melanoma
Source: Biomedicines. 2019 Nov 23;7(4):91. doi: 10.3390/biomedicines7040091 (PMC6966619; doi:10.3390/biomedicines7040091)
Supplement: Supplementary file 1 [file biomedicines-07-00091-s001.pdf]

Simplified Admix Archaeal Glycolipid Adjuvanted Vaccine and Checkpoint Inhibitor Therapy Combination Enhances Protection from Murine Melanoma

Felicity C. Stark, Gerard Agbayani, Jagdeep K. Sandhu, Bassel Akache, Charis McPherson, Lise Deschatelets, Renu Dudani, Melissa Hewitt, Yimei Jia, Lakshmi Krishnan and Michael J. McCluskie

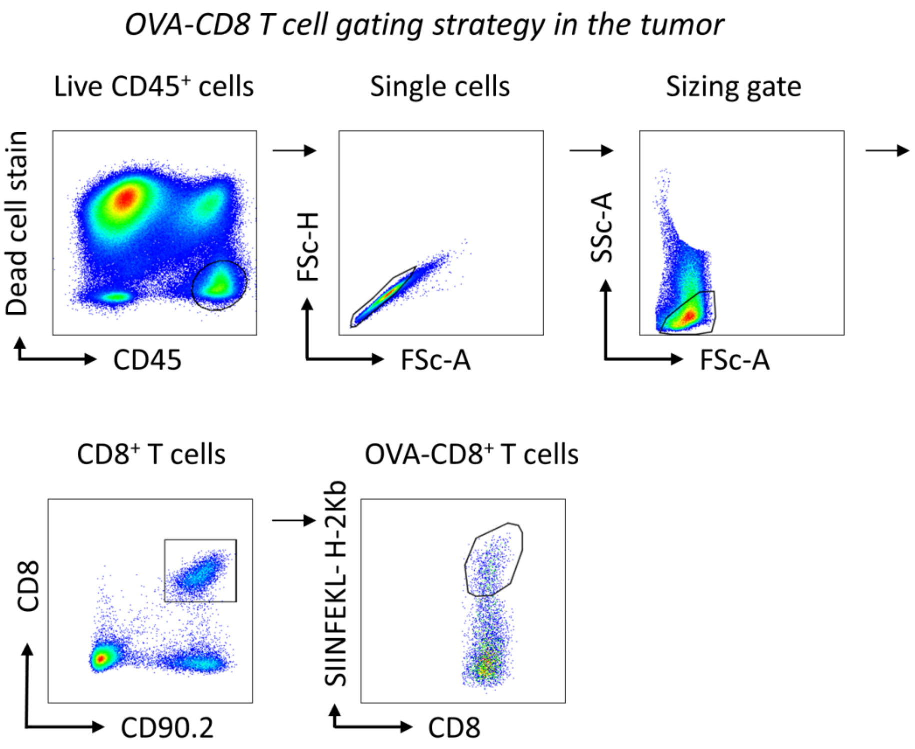

Figure S1. Frequency of OVA-CD8⁺ T cells in the spleen and tumor at the humane endpoint of C57BL/6 mice challenged with a solid melanoma tumor and treated with archaeosomes in combination with anti-PD-1 and anti-CTLA-4. C57BL/6 mice were given 5 x 10^5 B16-OVA tumor cells s.c. in the dorsal flank and treated as described in Figure 1. Tumor and spleens were harvested at the humane endpoint and OVA-CD8⁺ T cells were detected using flow cytometry. Using FlowJo, the flow cytometry gating strategy is shown to identify OVA-CD8⁺ T cells.

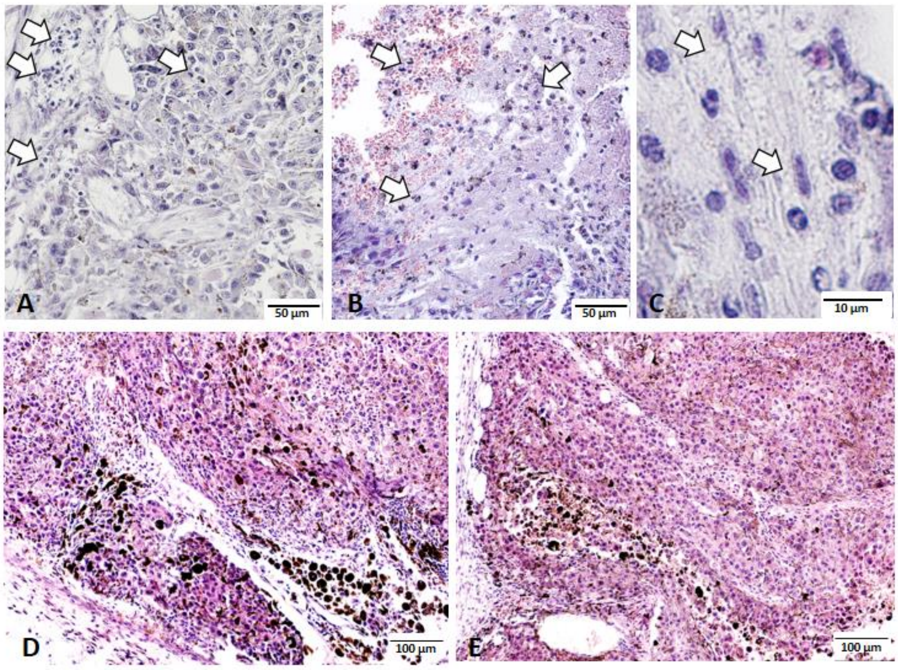

Figure S2. Hematoxylin and eosin stained photomicrographs of a B16-OVA tumor. Neutrophils were present at the tumor periphery (**A**, arrows) and around necrotic areas (**B**, arrows). A higher magnification image shows polymorphonuclear cells resembling neutrophils (**C**, arrows). B16-OVA tumor-bearing mice treated with CPI therapy (**D**) and CPI and SLA-OVA (**E**) showing increased tumor cell death (dark pigmented cells).
